# S06-1 Putting young people at the heart of physical activity research design: The Walking In ScHools (WISH) Study

**DOI:** 10.1093/eurpub/ckac093.028

**Published:** 2022-08-29

**Authors:** Marie H Murphy, Alison M Gallagher, Angela Carlin, S Maria O’Kane, Leanne C Doherty, Ian M Lahart, Russell Jago, Maria Faulkner

**Affiliations:** 1 Centre for Exercise Medicine, Physical Activity and Health, Sports and Exercise Sciences Research Institute, Ulster University, Jordanstown Campus, Newtownabbey BT37 0QB, UK; 2 Nutrition Innovation Centre for Food and Health (NICHE), Biomedical Sciences Research Institute, Ulster University, Coleraine Campus, Coleraine BT52 1SA, UK; 3 Faculty of Education, Health and Wellbeing, University of Wolverhampton, Walsall Campus, Gorway Road, Walsall WS1 3BD, UK; 4 Centre for Exercise, Nutrition & Health Sciences, School for Policy Studies, University of Bristol, Bristol BS8 1TZ, UK; 5 Department of Law and Humanities, Letterkenny Institute of Technology, Port Road, Letterkenny, Ireland

**Keywords:** Patient and Public Involvement, PPI, Young People, Physical Activity

## Abstract

**Background:**

Young people have the right to be informed and consulted about decisions affecting their lives. Youth Patient and Public Involvement (PPI) should be encouraged to ensure research is carried out ‘with' or ‘by' young people rather than ‘to', ‘about' or ‘for' them. PPI can ensure research is relevant, results are accessible and recruitment rates are improved. Young people have had limited involvement in the design, implementation and dissemination of public health research and there have been calls for a greater focus on youth PPI in research.

**Methods:**

Following the WISH feasibility study that consulted young people pre and post-intervention, a Youth Advisory Group (YAG) was set up within the main trial. The WISH study is a clustered randomised controlled trial in which a peer-led, school-based, brisk walking intervention is compared to usual physical activity in adolescent females. The YAG was introduced to inform intervention delivery and provide researchers with an understanding of what would encourage/discourage participation. Schools were asked to invite pupils aged 12-14 years (participants) and 15-18 years (walk leaders). Participative methods were used to develop and review study documentation. The YAG completed a short questionnaire and recruitment rates were monitored.

**Results:**

Fourteen pupils from 3 schools attended the 2019 YAG meeting. The YAG agreed the meeting was a good way of getting young people involved in research (93%) and attendees enjoyed the meeting (100%). As a result, changes were made to study documentation, incentives were purchased and recruitment materials developed. Participant recruitment was higher in schools who participated in the YAG (54%) compared to those who did not (47%). In 2021 the second YAG occurred and 1 teacher, 12 participants and 10 walk leaders from 2 schools provided feedback on the trials COVID-19 contingency plan. The girls felt their feedback was valued (100%) and it was important young people had the chance to contribute to research studies (100%).

**Conclusions:**

The views of young people have been central to the development of the WISH Study and although youth PPI is not without challenges, there are many benefits for researchers, the study and the young people involved.

